# Spatiotemporal pattern analysis of juglans leaf necrosis disease occurrence and development in southern Xinjiang, China, based on UAV

**DOI:** 10.3389/fpls.2025.1633206

**Published:** 2025-09-29

**Authors:** Heyu Zhang, Lei Guan, Zhaokun Geng, Xinglei Ma, Qiang Zhang, Baoqing Wang, Cuifang Zhang

**Affiliations:** ^1^ College of Forestry and Landscape Architecture, Xinjiang Agricultural University, Urumqi, China; ^2^ Xinjiang Academy of Forestry Sciences, Urumqi, China

**Keywords:** juglans leaf necrosis disease, unmanned aerial vehicle remote sensing, hyperspectral imagery, vegetation indices, disease severity classification, spatiotemporal analysis, precision agriculture, orchard disease monitoring

## Abstract

Juglans leaf necrosis (JLN) is a physiological disease primarily associated with abiotic stressors such as high temperatures, drought, and soil salinity, though biotic factors may also exacerbate its severity. It is a global concern affecting walnut production in multiple regions, including Xinjiang, China. In recent years, climate change, shifting agricultural practices, and disease transmission have increased its incidence, severely affecting tree growth, yield, and quality. Traditional field-based monitoring is labor-intensive and often inaccurate, underscoring the need for advanced remote sensing. To provide fast and objective monitoring, we used hyperspectral and high-resolution RGB imagery acquired by an unmanned aerial vehicle (UAV) to track JLN from June to September 2024 in southern Xinjiang. Five survey rounds captured the progression of disease severity. Among 17 vegetation indices, the modified red edge simple ratio (MRESRI), carotenoid reflectance index 1 (CRI1), and photochemical reflectance index (PRI) were the most informative for severity mapping. A Random Forest classifier achieved 86% overall accuracy and a Cohen’s kappa of 0.825. Spatial patterns showed persistent hotspots in low-lying areas, near roads, and in dense stands. These findings provide an effective, scalable approach for early detection and severity assessment, enabling timely, targeted interventions. Adoption of UAV-based hyperspectral monitoring can improve field surveillance, optimize resource allocation, and support sustainable walnut production.

## 1 Introduction

Juglans leaf necrosis (JLN) is a physiological disease primarily caused by abiotic factors that affect walnut yield and quality. This physiological disorder is caused, directly or indirectly, by unsuitable physical or chemical environmental factors, particularly environmental stressors such as high temperatures, water shortage, and soil salinity (Gao, 2017; Bai, 2022; Xing et al., 2023). These factors disrupt the tree’s water transport system, leading to characteristic symptoms such as browning, curling, and drying of the leaves. Early-stage symptoms manifest as tan focal spots on the leaf margins. As the disease progresses, these spots expand along the main veins, eventually leading to complete scorching of the leaf. In severe cases, nearly the entire canopy of a walnut tree turns brown (Xing et al., 2023). If not prevented and controlled, JLN will cause a decrease in leaf photosynthetic capacity, a reduction in individual fruit weight, and an increase in the rate of empty shells, ultimately leading to a significant decline in yield and quality and posing severe challenges to local farmers’ income growth and ecological economic development (Guo et al., 2024). In Xinjiang, walnuts occupy the largest orchard area of all economic tree crops, especially in the Aksu, Hotan, and Kashgar prefectures, where they provide more than half of farmers’ cash income and underpin local poverty-alleviation strategies (Zhang, 2011; Zhao et al., 2011; Huang, 2014). Beyond their economic role, walnut plantings help stabilize oasis ecosystems and combat desertification (Bai, 2022). Since its first report in Luopu County, Hotan Prefecture, in 2008, the disease has spread extensively across major walnut-producing areas in southern Xinjiang, including Aksu, Kashgar, and Hotan (Wu et al., 2021). Initially, because of the small scale of the walnut industry, the area and severity of JLN were relatively limited, and the impact on production was not significant, so it did not attract sufficient attention. However, as the scale of Xinjiang’s walnut industry has stabilized in recent years, JLN continues to occur in the main production areas of southern Xinjiang, and the area affected by JLN has shown an expanding trend, while related pests and diseases have also become prominent (Zhou, 2021). This disease has seriously hindered the healthy development of Xinjiang’s walnut industry and has become a major bottleneck. Research to date has centered on causal factors (Zhang et al., 2012; Liu, 2014; Wang et al., 2015; Tang and Hu, 2019), while quantitative, orchard-scale monitoring—especially using unmanned aerial vehicle (UAV) imagery—remains virtually unexplored. Developing such assessments is now critical for timely disease management and for safeguarding the economic and ecological value of Xinjiang’s walnut industry.

Traditionally, to accurately describe the types, severity, and symptoms of forest pests and diseases, time-consuming and labor-intensive field surveys are required in small sample areas. However, this method is limited and discontinuous in space; it is impossible to grasp the spatial distribution of pests and diseases in real time, which does not meet the needs of real-time monitoring, early warning, and forecasting of pests and diseases on a large regional scale. It has the disadvantages of being time-consuming and labor-intensive, insufficiently timely, low in accuracy, and susceptible to human errors (Mahanta et al., 2024). When pests and diseases occur on a large scale, it is difficult to carry out effective prevention and control due to subjective and untimely constraints (Saran et al., 2025). Real-time and efficient monitoring of the occurrence and dynamics of pests and diseases, along with timely and effective prevention and control, are key issues that need to be addressed in crop production to minimize the losses caused by pests and diseases. Recently, UAV-based remote sensing has emerged as a promising approach for detecting forest pests and diseases (Xiao et al., 2022; Du et al., 2024). Compared with traditional field monitoring, UAV remote sensing technology has the advantages of rapid data collection, wide spatial coverage, and low cost (Li et al., 2019; Zhao et al., 2023; Guo et al., 2023; Zhang and Zhu, 2023; Demir et al., 2024). The integration of UAV-based remote sensing in plant disease detection has garnered extensive acceptance, surpassing conventional manual detection methods (Bagheri, 2020; Sangaiah et al., 2024; Shan et al., 2024). By acquiring high temporal, spatial, and spectral resolution images, it can achieve large-scale pest and disease monitoring as well as early warning and forecasting (Backoulou et al., 2011; Duarte et al., 2022). The use of drones has greatly improved the level of modernization in agriculture and forestry and is of great significance to promoting the development of modern agriculture and forestry.

Hyperspectral remote sensing, a major advancement since the late 20th century, offers more spectral bands than multispectral systems, enabling more accurate target measurement and improved detection of forest pests and diseases (Liu et al., 2021; Feng et al., 2021; Yu et al., 2021; Iordache et al., 2020; Li et al., 2020; Wijesingha et al., 2021; Lin et al., 2021). Recent progress in unmanned aerial vehicle (UAV) technology has broadened its use in environmental monitoring and precision agriculture, with UAV-based image processing supporting autonomous decision-making (Turan et al., 2021) and photogrammetry enabling detailed surface models (Varol, 2025). Structure-from-Motion (SfM) has been applied for high-fidelity 3D reconstructions (Yakar and Dogan, 2018; Şasi and Yakar, 2017), and accuracy assessments confirm UAV model reliability with or without ground control points (Maraş and Nasery, 2023). Together, these advances provide a solid foundation for UAV-based hyperspectral imaging in detecting and monitoring juglans leaf necrosis (JLN), where high spatial resolution and temporal flexibility are essential. This technology has been widely used in precision agriculture, forestry, environmental protection, disaster monitoring, and early warning, and has broad potential for application (Kooistra et al., 2014). It has been widely used in apple mosaic virus disease (Jiang et al., 2023), wheat leaf rust (Terentev et al., 2023), and monitoring of rice leaf beetle infestation (Liu et al., 2020), showing its high value and broad application prospects in early disease monitoring. In recent years, the rapid development and application of hyperspectral remote sensing technology have provided unique spectral data for each pixel in hyperspectral images, compared with traditional field spectrometers, achieving the effect of “spectral-image fusion” (Zhang et al., 2023). The high spatiotemporal resolution of UAV hyperspectral imaging is a significant advantage, as it can be used to analyze the occurrence and development of JLN in the study area without being influenced by production conditions, geographical factors, or human subjectivity (Wang et al., 2023). This method can effectively monitor large affected areas with low operating costs, high efficiency, and good timeliness, greatly improving monitoring efficiency, reducing costs, and minimizing adverse ecological impacts. Monitoring of forest pests and diseases with UAV-borne hyperspectral imaging has so far centered on single-date acquisitions (Lausch et al., 2013; Li et al., 2020; Lin et al., 2021; Yu et al., 2021). By comparison, multi-temporal applications remain limited (Iordache et al., 2020; Einzmann et al., 2021; Bárta et al., 2022), despite their capacity to portray the complete infestation trajectory and pinpoint optimal detection windows.

In recent decades, climate change has emerged as a major driver intensifying abiotic stress in crops, especially in arid and semi-arid regions. Temperature extremes, altered precipitation patterns, and heightened evapotranspiration increase the frequency and severity of water scarcity events, directly impacting crop physiology. According to the Intergovernmental Panel on Climate Change (Shukla, 2019), these climatic changes have already reduced crop yields in many parts of the world through shortened maturation periods, reduced grain set during flowering, and increased water stress. Such stressors exacerbate the environmental conditions conducive to juglans leaf necrosis (JLN), leading to earlier symptom onset and more rapid disease progression. In southern Xinjiang, rising summer temperatures, reduced precipitation, and increasingly variable irrigation availability create an environment conducive to the development of JLN. In addition, shifts in agricultural practices, such as intensified monoculture systems and higher tree densities, can amplify microclimatic stress, further increasing disease risk. Recognizing these interactions is critical for designing adaptive disease monitoring and management strategies under a changing climate. In view of this, this study focuses on the main walnut variety “Zha 343” in Lop County, Hotan Prefecture. It calculates vegetation indices based on UAV hyperspectral images, identifies the most informative vegetation indices, evaluates plant health, classifies disease severity, captures the dynamic progression of JLN at different severity levels, and provides a preliminary analysis of its causes. This study aims to provide a more efficient, accurate, and real-time method to detect and map the spatial distribution of JLN so that timely intervention measures can be taken. By exploring the potential of UAV hyperspectral remote sensing technology in large-scale disease monitoring and integrated management strategies, it contributes to the sustainable development of Xinjiang’s walnut industry and provides a model for more effective agricultural disease management and ecological protection in the region and globally.

## 2 Materials and methods

### 2.1 Study area

**Figure 1:**
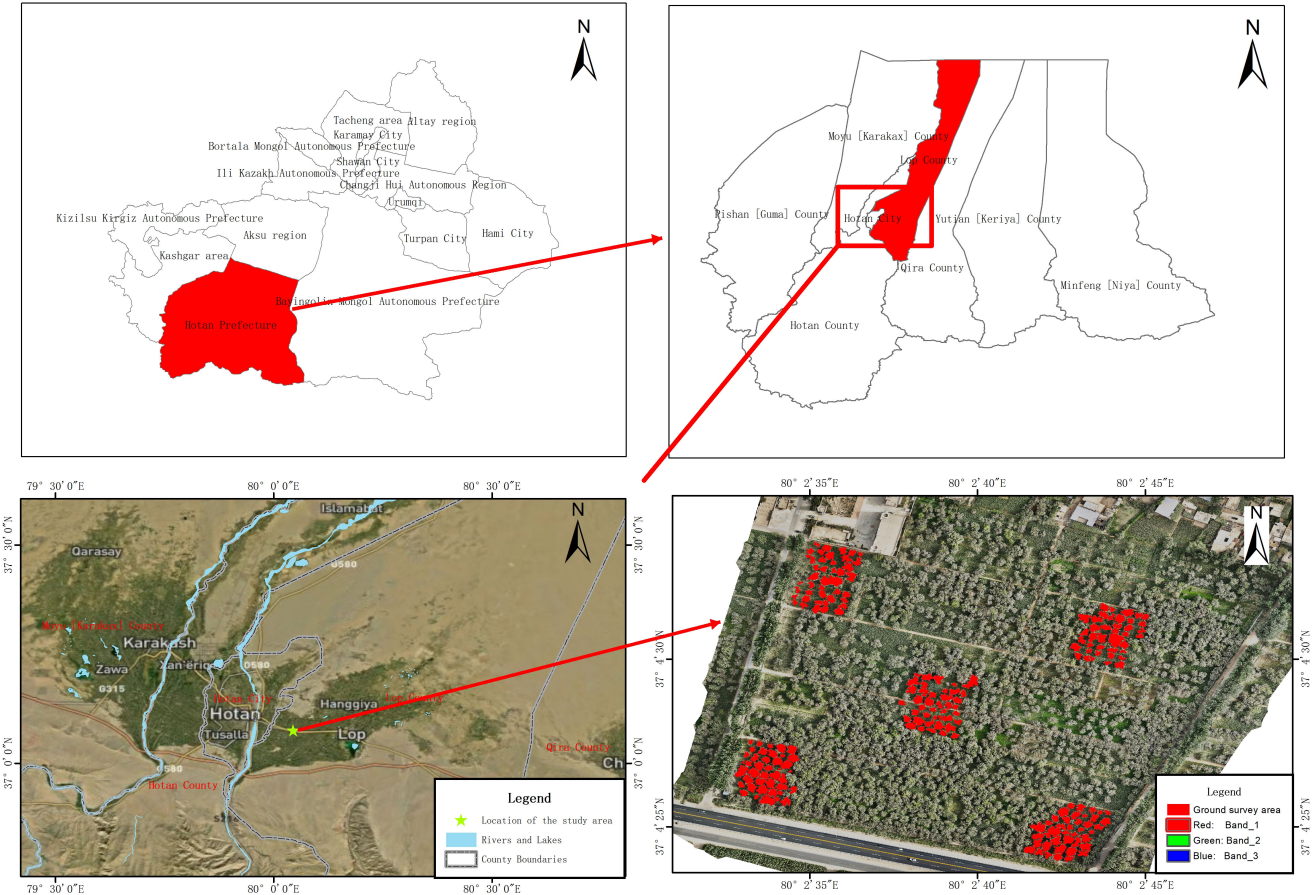
Geographical location map of the study area. **(A)** shows the location of Hotan Prefecture, **(B)** shows the location of Luopu County, Hotan Prefecture, **(C)** is a satellite image of the location of the research area, and **(D)** shows the Drone image and survey area in the study area.

Luopu County, administered by Hotan Prefecture in the Xinjiang Uygur Autonomous Region, lies along the northern foothills of the Kunlun Mountains on the southern fringe of the Tarim Basin, where an extremely arid continental climate prevails, marked by large diurnal temperature variations, low humidity, annual evaporation of 2,226.2 mm, and frequent dust-laden winds (Liang et al., 2005; Liu and Wei, 2005; Xu et al., 2009). Mean annual temperature ranges from 7.8°C to 12°C (extremes: –24.6°C to 40.1°C), while average annual precipitation is only 35.2 mm. The study was conducted in an 8-ha orchard in Kuochaiker Aizik Village, Luopu County (**Figure 1**), planted mainly with the ‘Zha 343’ walnut cultivar, with a smaller proportion of ‘Xinfeng’. Approximately 70% of trees are 13 years old, 20% are 7–13 years old, and 10% are younger than 7 years, at a spacing of 6 m × 8 m.

### 2.2 Ground survey

To comprehensively investigate the progression of juglans leaf necrosis (JLN) at different growth stages, a series of UAV data acquisitions and ground surveys were conducted under clear, light-wind conditions on five key dates: June 16, July 2, July 30, August 12, and August 30, 2024. These time points were selected to capture critical changes during the growing season. Special emphasis was placed on the peak disease period typically observed in early July, thereby enabling a comprehensive assessment of JLN onset, severity, and subsequent decline under field conditions.

**Figure 2:**
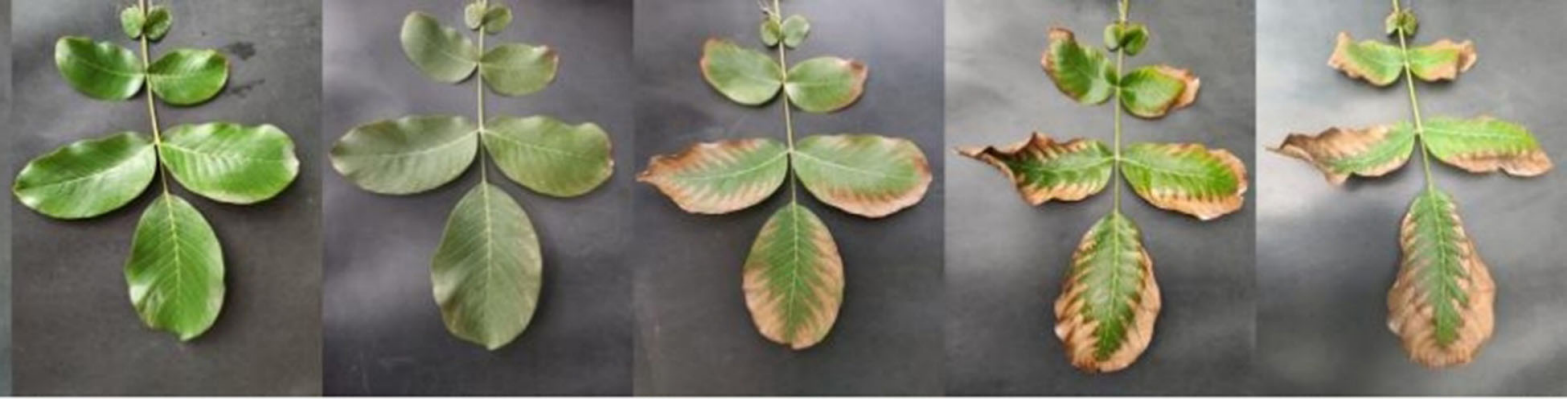
juglans leaf necrosis disease occurrence in different periods. The image is from the author’s photography of the extent of leaf disease in the experimental field.


During each survey, we selected five disease plots, with 40 trees sampled from each plot (**Figure 1D**). To account for potential spatial variability within the canopy, leaves were collected from five canopy positions—east, south, west, north, and center—with three leaves per position, totaling 15 leaves per tree. Subsequently, the disease severity of these 15 leaves was evaluated to comprehensively assess the prevalence and severity of the disease across the entire canopy. The classification criteria for leaf-level disease severity are detailed in **Table 1**. Photographs of diseased leaves at different severity levels are shown in **Figure 2**. The calculation formula for leaf disease severity is presented in Equation 1.

**Table 1:** Leaf disease rating criteria.

Disease Grade	Description
Grade 0	No signs of browning or scorch on the leaf surface.
Grade 1	Browning/scorch affects ≤ 25% of the leaf surface.
Grade 2	Browning/scorch affects 26%-50% of the leaf surface.
Grade 3	Browning/scorch affects 51% -75% of the leaf surface.
Grade 4	Browning/scorch affects 76% -100% of the leaf surface.

(1) $$\text{Leaf severity}(\%)=\frac{\text{Leaf spot area}}{\text{Total leaf area}}\times 100$$

### 2.3 UAV data collection

#### 2.3.1 Visible light high-resolution imaging

High-resolution visible imagery was acquired using the DJI Mavic 3E (DJI, Shenzhen, China; https://www.dji.com) UAV equipped with a standard RGB sensor. Missions were conducted at an altitude of 80 m to achieve the best balance between area coverage and spatial resolution. To minimize atmospheric and illumination variability, all flights were performed between 10:00 and 12:00 local time under clear-sky or lightly clouded conditions, avoiding strong wind events (>5 m/s). Data were collected on five survey dates (June, July, August, early September, and late September 2024) to capture disease progression. Forward overlap and side overlap were set at 80% and 70%, respectively, to ensure accurate image stitching without gaps. High-resolution visible imagery was processed and stitched using DJI Terra software (DJI, Shenzhen, China; https://www.dji.com/dji-terra), applying structure-from-motion principles to produce orthophotos with a spatial resolution of approximately 2.2 cm.

#### 2.3.2 Hyperspectral imaging

Hyperspectral data were acquired using a DJI M350 (DJI, Shenzhen, China; https://www.dji.com) equipped with an FS-60c hyperspectral sensor (Hangzhou CHNSpec Technology Co., Ltd, Hangzhou, China; https://www.ceseyi.com/aboutus.html). Flights were performed between 10:00 and 12:00 local time under clear-sky or lightly clouded conditions, avoiding strong wind events (>5 m/s), with a flight altitude of 120 m. Data were collected on five survey dates (June, July, August, early September, and late September 2024). Forward overlap and side overlap were set to 80% and 70%, respectively, to ensure full coverage. Prior to each flight, a standardized white reference panel was used for radiometric calibration. This acquisition protocol minimized environmental variability and ensured comparability across temporal datasets. The hyperspectral data from this sensor cover a spectral range of 400–1000 nm, with a spectral resolution of 2.17 nm and a spatial resolution of 11 cm. Data stitching and processing were conducted in FigSpec Studio software (Hangzhou CHNSpec Technology Co., Ltd, Hanzhou, China; https://www.ceseyi.com/aboutus.html), specific to the FS-60c sensor.

#### 2.3.3 Data preprocessing

**Figure 3:**
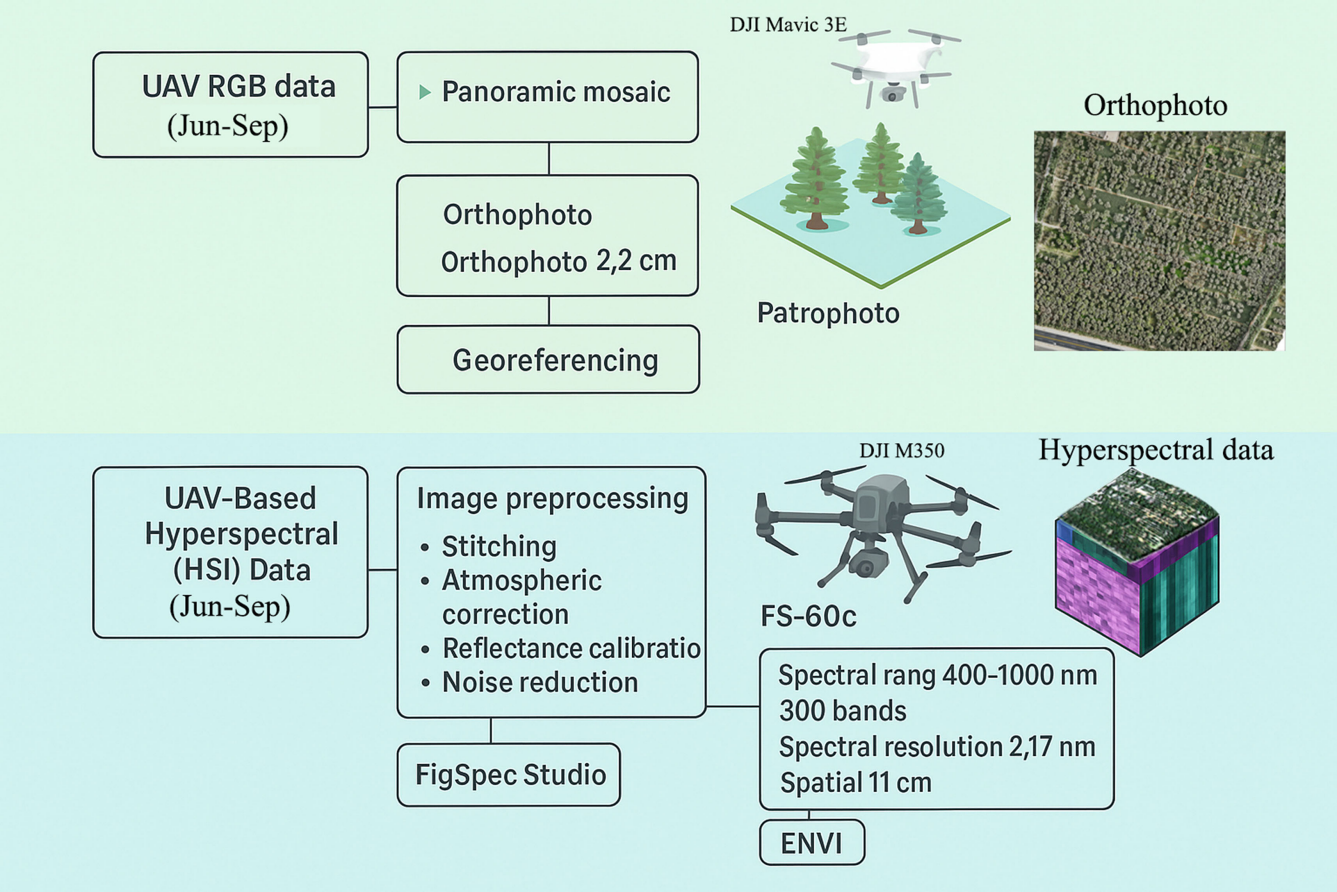
UAV data processing flow chart. Workflow for drone-acquired hyperspectral and RGB imagery processing used in this study. The process includes image stitching to generate seamless orthomosaics, atmospheric correction to remove atmospheric effects, reflectance calibration using reference panels to standardize spectral values, and noise reduction to improve data clarity. Preprocessed imagery is then used to calculate vegetation indices (VIs), such as NDVI (Normalized Difference Vegetation Index), MRESRI (Modified Red-Edge Simple Ratio Index), CRI1 (Carotenoid Reflectance Index 1), and PRI (Photochemical Reflectance Index). These indices are integrated into a Random Forest (RF) model for disease severity classification. Spatial analyses, including mapping and area statistics, are conducted using a Digital Elevation Model (DEM) and ArcGIS 10.8. Abbreviations: DEM, Digital Elevation Model; NDVI, Normalized Difference Vegetation Index; VI, Vegetation Index; RF, Random Forest; MRESRI, Modified Red-Edge Simple Ratio Index; CRI1, Carotenoid Reflectance Index 1; PRI, Photochemical Reflectance Index.

Hyperspectral data preprocessing included stitching, atmospheric correction, reflectance calibration, and noise reduction. Stitching was used to combine multiple flight images into a seamless multi-band dataset; atmospheric correction minimized the impact of atmospheric interference; reflectance calibration ensured the consistency of spectral values using gray reference panels; and noise reduction eliminated irrelevant signals and improved clarity. Stitching, atmospheric correction, and reflectance calibration were performed in FigSpec Studio, while noise reduction was conducted in ENVI. The preprocessed datasets formed the basis for vegetation index calculations and dynamic analysis of JLN. A supervised classification approach was employed to identify the presence and severity of walnut juglans leaf necrosis (JLN). The UAV data processing flow is shown in **Figure 3**.

### 2.4 Classification methodology

We use the random forest algorithm to classify the severity of juglans leaf necrosis. The basic principle of this method was to extract and analyze spectral features related to canopy plant stress (Tuominen et al., 2008). Feature selection was first performed to identify vegetation indices sensitive to plant health change. Then, a Random Forest classifier was trained on labeled examples of JLN severity, and its aggregated predictions produced a robust classification (Vanguri et al., 2024; Yang et al., 2015). The RF classifier was implemented in ENVI with 500 trees, a maximum tree depth of 20, bootstrap sampling enabled, and Gini impurity as the splitting criterion. Class boundaries for severity levels were not manually set; instead, they were determined by the RF’s decision tree splits, ensuring a data-driven thresholding process. The performance of the Random Forest model was validated using a confusion matrix and a suite of evaluation indicators—including accuracy, precision, and recall—based on a sample of JLN severity data from ground surveys (Rissati et al., 2020). After classification, the results were refined to eliminate obvious errors and exclude patches located outside the walnut canopy. The severity of JLN was divided into five levels: Grade 0 (healthy) indicates no symptoms; Grade 1 (mild) indicates mild damage; Grade 2 (moderate) indicates larger, more continuous patches of leaf damage.; Grade 3 (severe) indicates severe crown damage; and Grade 4 (critical) indicates that most of the crown was affected (Wang and Jin, 2023). The classification standards are shown in **Table 1**. To validate UAV-based disease detection, we conducted systematic ground surveys concurrent with UAV flights. A total of 1,000 georeferenced sampling points were established across the study orchards, ensuring representation of all disease severity levels (Grade 0–4). At each point, three to five walnut trees were visually assessed using the standardized juglans leaf necrosis (JLN) severity scale. Symptomatic and asymptomatic trees were recorded, and severity grades were assigned based on leaf chlorosis, necrosis percentage, and canopy damage patterns. All ground samples were geotagged using a sub-meter accuracy GNSS receiver and spatially matched to UAV-derived hyperspectral and RGB orthomosaics. The 1,000 samples were randomly split into a training set (80%) and validation set (20%) for supervised classification. Spatial and statistical analyses were conducted in ArcGIS 10.8 (Environmental Systems Research Institute, Inc., Redlands, CA, USA; https://www.esri.com)

### 2.5 Accuracy assessment

To evaluate classification performance, we used a confusion matrix along with Overall Accuracy (OA), accuracy, precision, recall, and Cohen’s kappa coefficient (*k*). Cohen’s kappa provides a chance-corrected measure of agreement between the classified map and reference data. It is calculated as:


$$k=\frac{\rho_{o}-\rho_{e}}{1-\rho_{e}}$$

where $\rho_{o}\;\;$ is the observed agreement and $\rho_{e}$ is the expected agreement by chance. The interpretation of *k* follows Landis and Koch (1977): values <0.00 = poor; 0.00–0.20 = slight; 0.21–0.40 = fair; 0.41–0.60 = moderate; 0.61–0.80 = substantial; and 0.81–1.00 = almost perfect agreement.

### 2.6 Vegetation indices

Hyperspectral data were processed in ENVI software (Exelis Visual Information Solutions, USA; https://envi.geoscene.cn/), and three types of indices were derived: greenness indices, pigment indices, and canopy water/photochemical efficiency indices. The greenness indices primarily reflect vegetation vigor by detecting variations in reflectance between red and near-infrared (NIR) bands, with healthy vegetation typically exhibiting higher values; we calculated nine such greenness indices, including NDVI, EVI, and various red-edge indices, as listed in **Table 2** (Galvão et al., 2013; Liu et al., 2007). The leaf pigment indices, which depends on reflectance in the green and red bands, estimates leaf pigment content (especially chlorophyll) closely linked to photosynthetic performance and overall plant vitality; this study determined four pigment-related indices to provide insights into the nutritional status and health of walnut trees, also shown in **Table 2** (Ahmad et al., 2020; Velichkova and Krezhova, 2018). Meanwhile, the canopy water or photosynthetic efficiency indices characterize vegetation water content and photosynthetic capacity, offering critical understanding of plant resilience under stress; here, four such indices were computed to assess potential water deficits and photosynthetic function decline in the orchards, as indicated in **Table 2** (Oliveira et al., 2019; Lu et al., 2015).

**Table 2:** The Vegetation indices calculated in the article.

Index Name	Formula	Reference
Normalized Difference Vegetation Index (NDVI)	$NDVI=\frac{\rho_{800}-\rho_{680}}{\rho_{800}+\rho_{680}}$	Rouse et al., 1973
Plant Senescence Reflectance Index (PSRI)	$PSRI=\frac{\rho_{680}-\rho_{500}}{\rho_{750}}$	Merzlyak et al., 1999
Enhanced Vegetation Index (EVI)	EVI=2.5·NIR−RedNIR+6·Red−7.5·Blue+1	Huete et al., 2002
Atmospherically Resistant Vegetation Index (ARVI)	$ARVI=\frac{\rho_{800}-[\rho_{680}-\gamma (\rho_{450}-\rho_{680})]}{\rho_{800}+[\rho_{680}-\gamma (\rho_{450}-\rho_{680})]}$	Kaufman and Tanre, 1992
Red Edge Normalized Difference Vegetation Index (RENNDI)	$\text{RENNDI}=\frac{\rho_{750}-\rho_{705}}{\rho_{750}+\rho_{705}}$	Sims and Gamon, 2002
Modified Red Edge Simple Ratio (MRESR)	$\text{MRESR}=\frac{\rho_{750}-\rho_{445}}{\rho_{705}-\rho_{445}}$	Sims and Gamon, 2002
Modified Red Edge Normalized Difference Vegetation Index (MRENDVI)	$\text{MRENDVI}=\frac{\rho_{750}-\rho_{705}}{\rho_{750}-\rho_{705}-2*\rho_{445}}$	Sims and Gamon, 2002
Vogelmann Red Edge Index 1 (VREI1)	$VREI1=\frac{\rho_{740}}{\rho_{720}}$	Vogelmann et al., 1993
Vogelmann Red Edge Index 2 (VREI2)	$VREI2=\frac{\rho_{734}-\rho_{747}}{\rho_{715}+\rho_{726}}$	Vogelmann et al., 1993
Carotenoid Reflectance Index 1 (CRI1)	$CRI1=\frac{1}{\rho_{510}}-\frac{1}{\rho_{550}}$	Gitelson et al., 2002
Carotenoid Reflectance Index 2 (CRI2)	$CRI2=\frac{1}{\rho_{510}}-\frac{1}{\rho_{700}}$	Gitelson et al., 2002
Anthocyanin Reflectance Index 1 (ARI1)	$ARI1=\frac{1}{\rho_{550}}-\frac{1}{\rho_{700}}$	Gitelson et al., 2001
Anthocyanin Reflectance Index 2 (ARI2)	$ARI2=\rho_{800}(\frac{1}{\rho_{550}}-\frac{1}{\rho_{770}})$	Gitelson et al., 2001
Water Band Index (WBI)	$\text{WBI}=\frac{\rho_{970}}{\rho_{900}}$	Peñuelas et al., 1993
Photochemical Reflectance Index (PRI)	$PIR=\frac{\rho_{531}-\rho_{570}}{\rho_{531}+\rho_{570}}$	Gamon et al., 1997
Structure Insensitive Pigment Index (SIPI)	$SIPI=\frac{\rho_{800}-\rho_{445}}{\rho_{800}+\rho_{445}}$	Penuelas et al., 1995
Red Green Ratio Index (RGRI)	$\text{RGRI}=\frac{\sum_{\text{i}=600}^{699}{\text{R}}_{\text{i}}}{\sum_{\text{j}=500}^{599}{\text{R}}_{\text{j}}}$	Gamon and Surfus, 1999

## 3 Results

### 3.1 Visible light high-resolution imaging analysis

**Figure 4:**
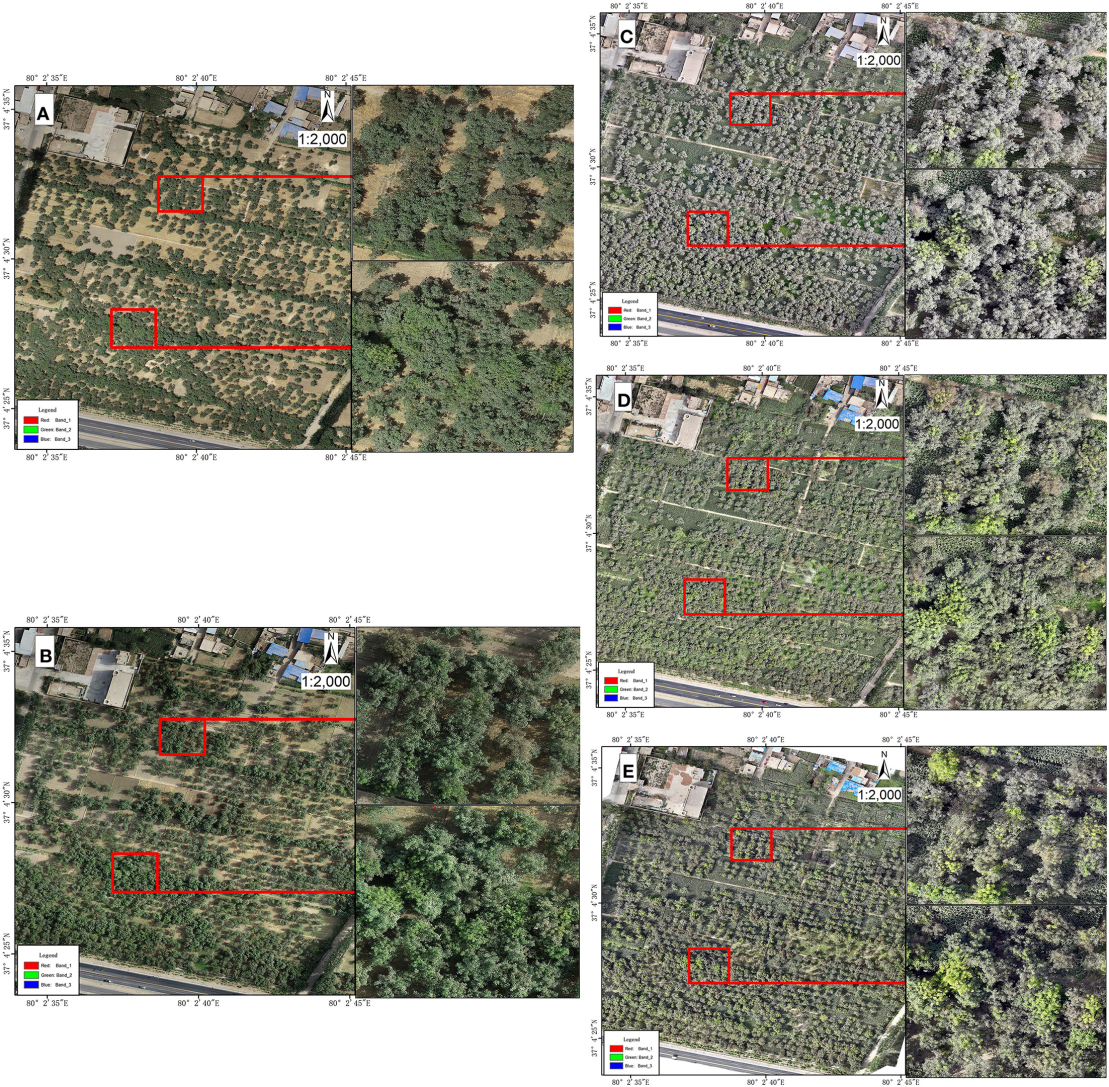
High-resolution visible light imaging results of experimental plots from UAVs. **(A)** shows the first stage of the experiment, **(B)** shows the second stage of the experiment, **(C)** shows the third stage of the experiment, **(D)** shows the fourth stage of the experiment, and **(E)** shows the fifth stage of the experiment. The right side of the picture is a partially enlarged image of the drone image.

When analyzing five stages of high-resolution UAV images of juglans leaf necrosis (JLN) in the experimental orchard, the dynamic changes in its occurrence and evolution could be clearly observed (**Figure 4**). In the first and second stages, JLN had just occurred, and symptoms were not obvious. From locally magnified UAV images, the lesions did not exhibit obvious color or texture changes and appeared mainly healthy green (**Figures 4A, B**). This may be because the leaf damage area was still small in the early stage, and spectral reflectance characteristics had not yet changed significantly. As time passed to the third experimental stage, symptoms were significantly aggravated, and affected leaves showed large damaged area. High-resolution images revealed marked changes in the walnut crowns, manifested as dry leaves and decreased reflectivity in diseased parts, especially in the visible band. From the locally enlarged image at Stage 3 (**Figure 4C**), crowns displayed a grayish-white appearance, which contrasted with Stages 1 and 2. This change in texture and color provided a reliable remote sensing indicator for JLN.

In the fourth and fifth stages, the degree of juglans leaf necrosis was further aggravated. Large areas of leaves showed scorch characteristics, and the diseased area expanded features. During this period, the spatial distribution of disease features became clearer. In addition, some walnut trees were observed to have begun to sprout new leaves. This may be due to the recovery growth after infection. As a result, images displayed the coexistence of new and dead leaves, creating complex spectral mixing in multispectral imagery (**Figures 4D, E**).

From the analysis results, we found that the complexity of detecting JLN increases as the disease progresses. Specifically, in the early stage of the disease, symptoms were subtle and difficult to distinguish from healthy crowns in RGB UAV imagery. In later stage, due to the recovery of the walnut itself, newly grown leaves may mask the infection characteristics, making field-based assessments and traditional RGB remote sensing increasingly unreliable. Therefore, more advanced technologies are needed to more accurately detect and diagnose the occurrence and development of juglans leaf necrosis.

### 3.2 Hyperspectral imagery classification results

**Figure 5:**
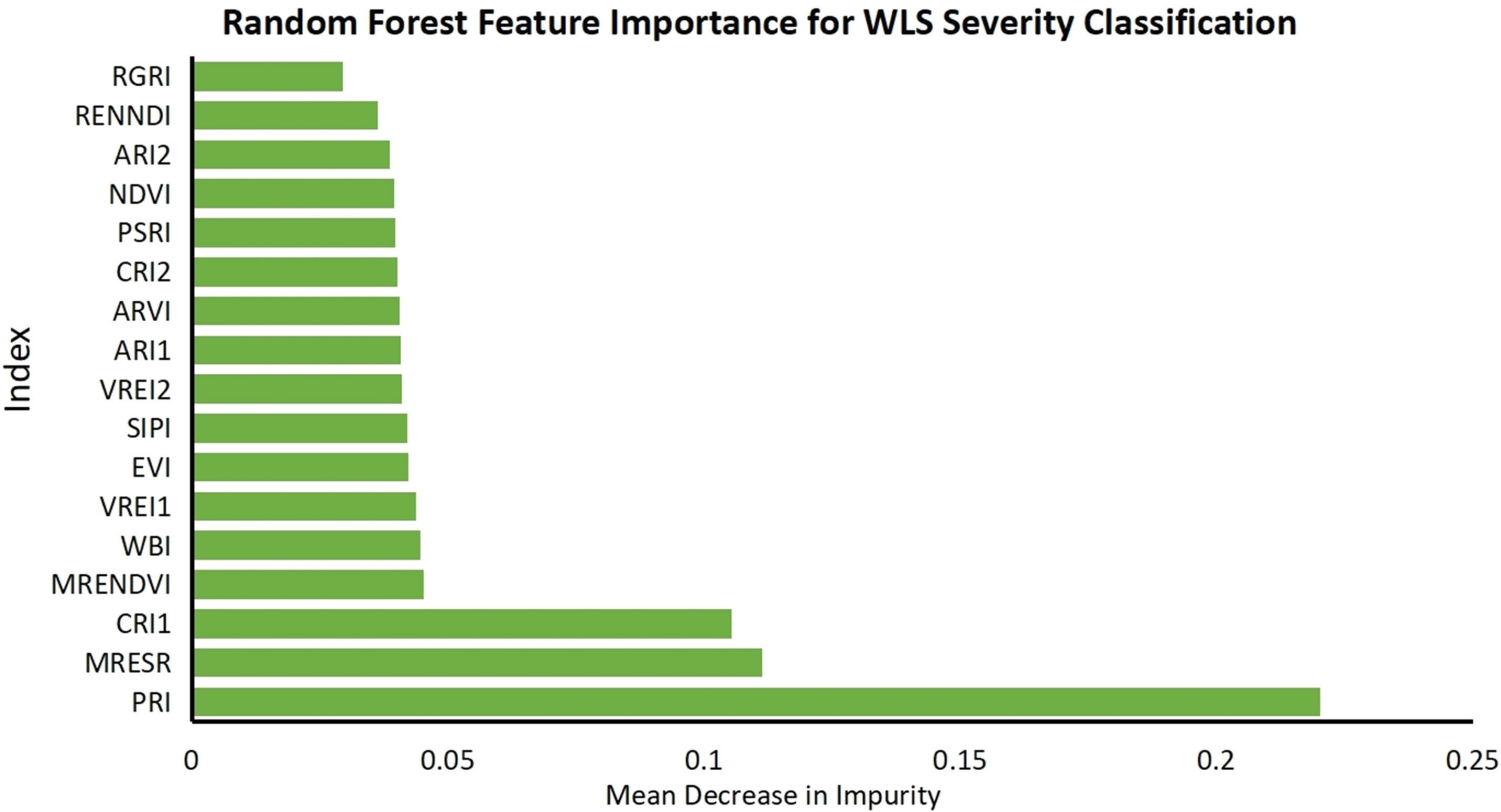
Random Forest feature importance for JLN severity classification. Relative importance of 17 vegetation indices (VIs) for differentiating juglans leaf necrosis (JLN) severity levels based on UAV hyperspectral imagery. Higher values indicate stronger predictive contribution to model decision-making. The specific meanings of the abbreviations are shown in **Table 2**.

To rigorously validate UAV-derived hyperspectral classification of juglans leaf necrosis (JLN) severity, we conducted five dedicated field campaigns contemporaneous with flight missions. A stratified sampling design yielded 1000 Geo-referenced crown centers, where visual assessments followed the national JLN severity standard (grade 0–4). For every sample point reflectances corresponding to the 17 pre-selected vegetation indices (VIs) were extracted from the atmospherically and radiometrically corrected hyperspectral orthomosaic. The random forest algorithm was used to calculate the importance of each VI for JLN severity (**Figure 5**). As can be seen from **Figure 5**, MRESRI, CRI1 and PRI clearly dominated the feature-importance ranking, indicating strong sensitivity of red-edge and carotenoid-related metrics to JLN stress. In contrast, indices such as MRENDVI, WBI and VREI1 contribute marginally, suggesting limited additional discriminatory power once these indices are included.

**Figure 6:**
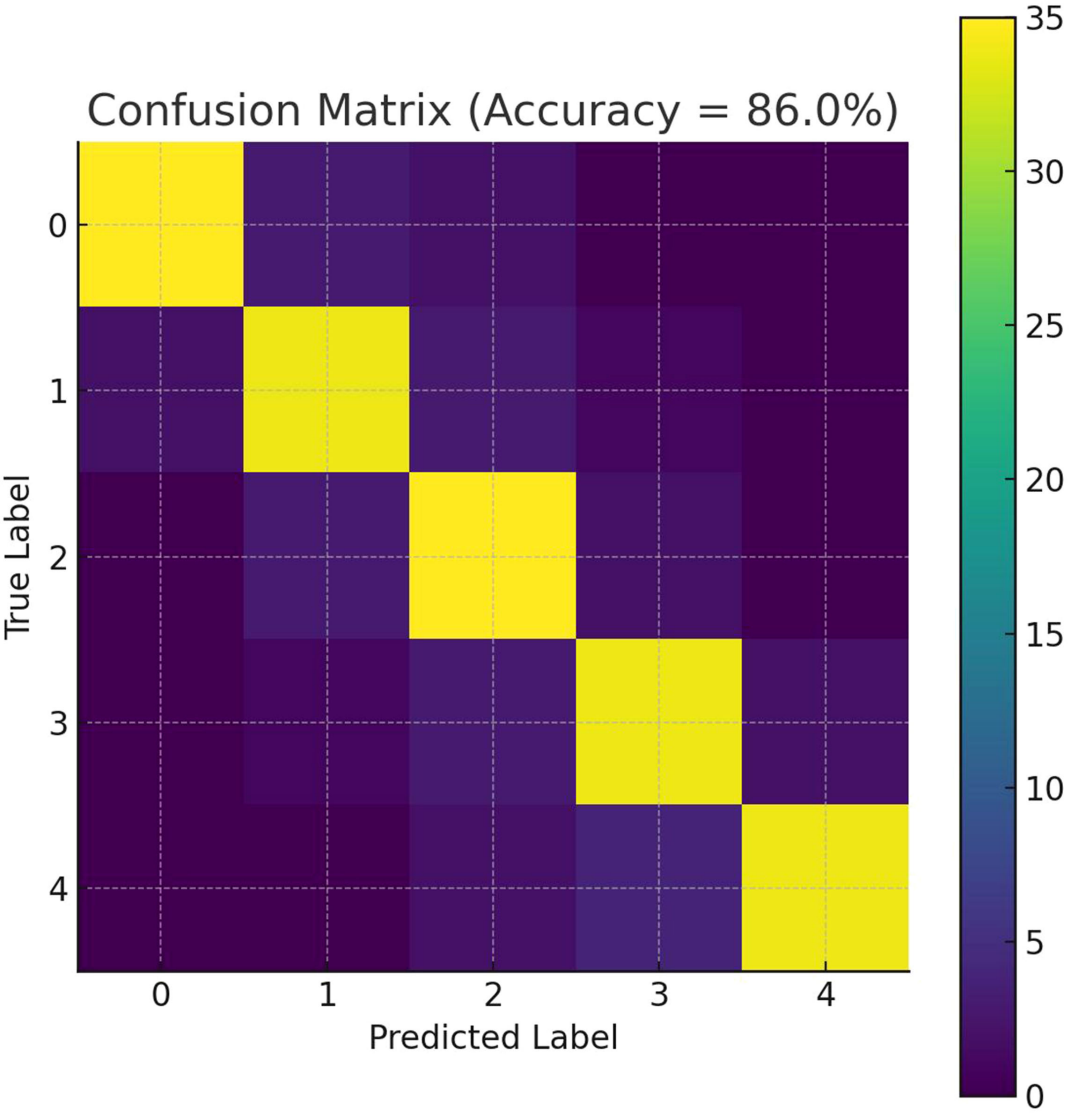
Confusion matrix heat map. Rows represent ground-truth classes and columns represent predicted classes. Values along the diagonal indicate correct classifications (true positives); off-diagonal values represent misclassifications. OA, Overall Accuracy; *k*, Cohen’s kappa coefficient.

The confusion matrix (**Figure 6**) demonstrated an overall accuracy (OA) of 86% (172/200) and a Cohen’s k of 0.825, denoting substantial agreement between RF predictions and ground observations. **Table 3** details class-wise statistics. Grades 0 and 4 achieved both high precision (≤ 6% false alarm) and high recall (≥ 85%), indicating that healthy and severely stressed crowns were rarely confused with other. Most of the 28 errors occurred between adjacent intermediate grades (1, 2 ↔ ↔, 3), which is expected given overlapping visual and spectral cues. Among them, Grade 1 had the lowest classification accuracy. Importantly, no misjudgments across more than two grades (such as 0→3 or 1→4), confirming reasonable decision boundaries. From the overall indicators (**Table 4**), balanced/macro average recall was 0.86, macro precision was 0.865, and Macro F1-score was 0.861, further confirm the balanced performance of the model across all severity stages. The high OA, robust *k* statistic and biologically coherent error structure indicated that the VI-based RF model was well suited for operational mapping of JLN severity. In particular, the dominance of red-edge and pigment-related indices highlighted their value as early-warning proxies for canopy stress.

**Table 3:** Precision analysis of severity of juglans leaf necrosis in various categories.

Severity class	TP	FN	FP	Precision	Recall	F-score	Main confusions (>1)
0 (healthy)	35	5	2	0.946	0.875	0.909	3 × →1, 2 × →2
1 (mild)	35	5	10	0.778	0.875	0.824	3 × ←0, 3 × →0, 2 × →2
2 (moderate)	34	6	7	0.829	0.850	0.840	3 × →3, 2 × ←1
3 (heavy)	34	6	6	0.829	0.850	0.840	3 × ←2, 2 × →4
4 (severe)	34	6	2	0.944	0.850	0.895	4 × ←3, 2 × ←2

TP, True Positive; FP, False Positive; FN, False Negative

**Table 4:** Overall metrics of the model.

Measure	Value
Overall accuracy	86% (172/200)
Balanced/macro-average recall	0.86
Macro precision	0.865
Macro F1-score	0.861
Cohen’s κ	0.825 (substantial agreement)

**Figure 7:**
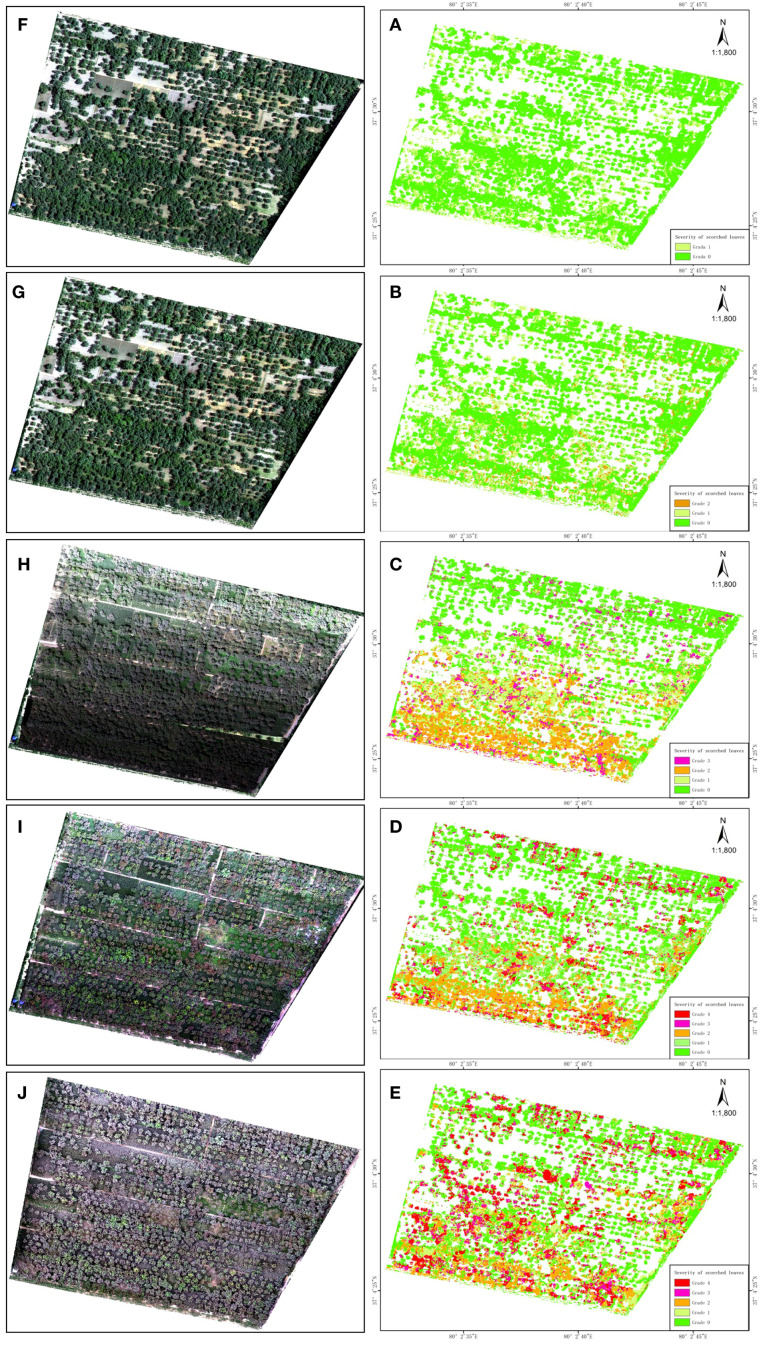
Classification results of UAV hyperspectral images of the experimental plot. **(A, E)** are the hyperspectral color composite images and classification results of the first stage of the experiment, **(B, F)** are the hyperspectral color composite images and classification results of the second stage of the experiment, **(C, G)** are the hyperspectral color composite images and classification results of the third stage of the experiment, **(D, H)** are the hyperspectral color composite images and classification results of the fourth stage of the experiment, and **(E, I)** are the hyperspectral color composite images and classification results of the fifth stage of the experiment.

The classification results are shown in **Figure 7**. From a temporal and spatial perspective, the classification results showed a clear trend in the occurrence and development of juglans leaf necrosis (JLN) in the experimental orchard, on which more detailed studies can be conducted to better understand the dynamics of the disease. The results also showed that it was feasible to calculate vegetation indices based on drone hyperspectral remote sensing data and used the random forest method to classify the severity of juglans leaf necrosis. The classification results of this method can fully help to understand the law of JLN spread and aggravation over time, as well as its spatial distribution evolution in different areas of the orchard.

### 3.3 Spatio-temporal dynamics of juglans leaf necrosis

**Figure 8:**
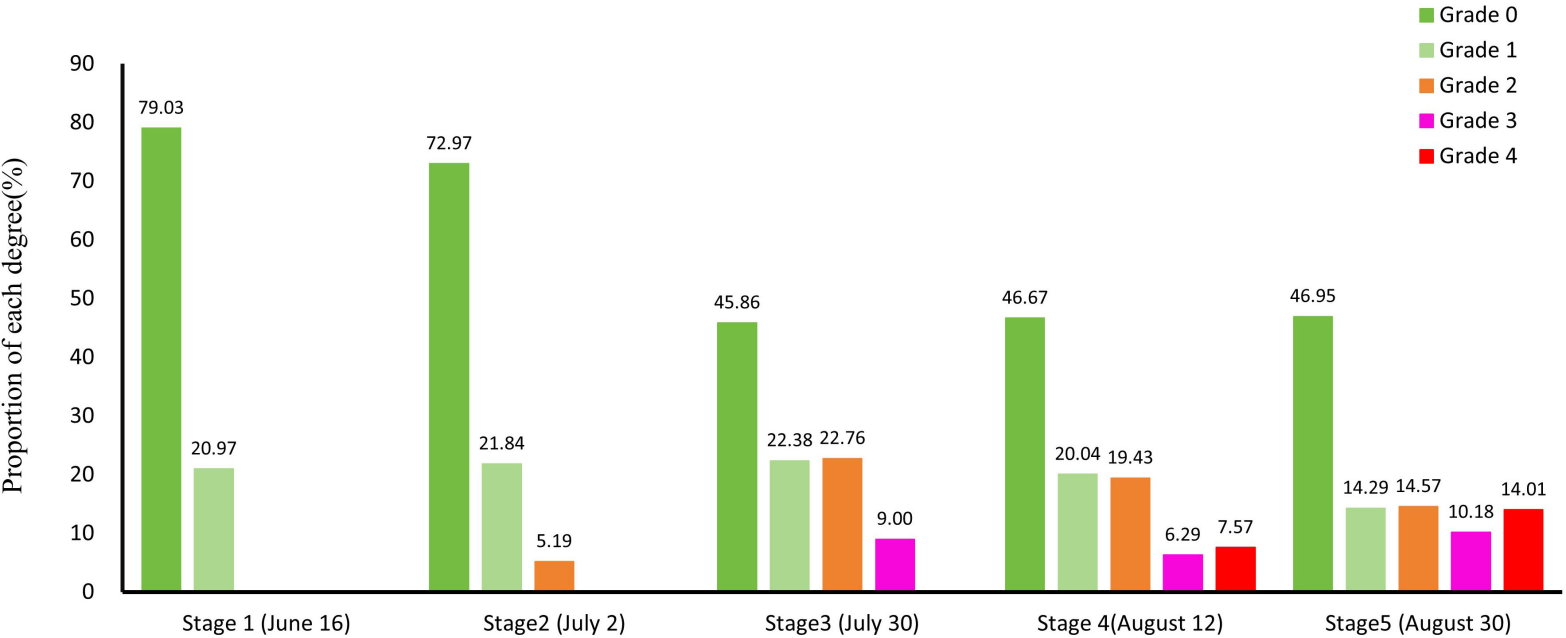
Statistical results of the severity of juglans leaf necrosis in different time periods. The X-axis shows monitoring stages (T1–T5), and the Y-axis shows the percentage of canopy pixels in each severity grade (Grade 0–4). Grades are color-coded: Grade 0 = healthy; Grade 1 = mild; Grade 2 = moderate; Grade 3 = severe; Grade 4 = critical.

**Figure 7** shows the hyperspectral classification maps, while **Figure 8** provides class-area statistics. Together, they show a clear escalation of JLN across the five monitoring stages.

#### 3.3.1 Stage 1

JLN was largely absent: 79.03 % of canopy pixels were healthy (grade 0) and 20.97 % showed only mild symptoms (grade 1). Diseased trees appeared as a few, randomly distributed patches, and no spatial pattern could yet be linked to topography or management factors.

#### 3.3.2 Stage 2

Two weeks later, grade 2 (moderate) pixels appeared (5.19 %), although grades 0 and 1 still dominated (72.97 % and 21.84 %, respectively). New foci were still small and scattered, indicating an incipient but spatially unconstrained spread.

#### 3.3.3 Stage 3

Disease intensity and aggregation increased markedly. Moderate damage (grade 2) expanded to 22.76 % of the orchard, and severe damage (grade 3) emerged (9 %). Most grade 2 and 3 pixels clustered in the southern sector, adjacent to the He–Luo Expressway and areas of high tree density—suggesting that road proximity and stand structure facilitate disease transmission.

#### 3.3.4 Stage 4

JLN reached its first peak. Although nearly half of the canopy remained healthy (46.67 %), grades 2–4 together accounted for 33.29 % (grade 2 = 19.43 %, grade 3 = 6.29 %, grade 4 = 7.57 %). Large, contiguous blocks of brown, desiccated foliage were evident in both the hyperspectral maps and the corroborating RGB−drone imagery, with severity highest where airflow and drainage are poorest.

#### 3.3.5 Stage 5

The final survey confirmed further intensification: grade 4 rose to 14.01 % and grade 3 to 10.18 %, while grades 1 and 2 declined to 14.29 % and 14.57 %, respectively. Despite widespread damage, pockets of regreening appeared, implying that micro−site factors (better ventilation, soil moisture, or nutrient status) confer partial resilience.

Overall, JLN advanced from isolated, mild lesions to extensive crown scorch within roughly six weeks. Spatial progression was initially random but became strongly clustered near highways and in dense stands, pointing to anthropogenic vectors and canopy microclimate as key drivers. The combined spatial and temporal evidence highlights the narrow window for effective intervention—between the first detectable grade 2 patches (Stage 2) and the rapid coalescence of severe foci (Stage 3)—before irreversible canopy loss ensues.

### 3.4 Transfer analysis between different severity levels

**Figure 9:**
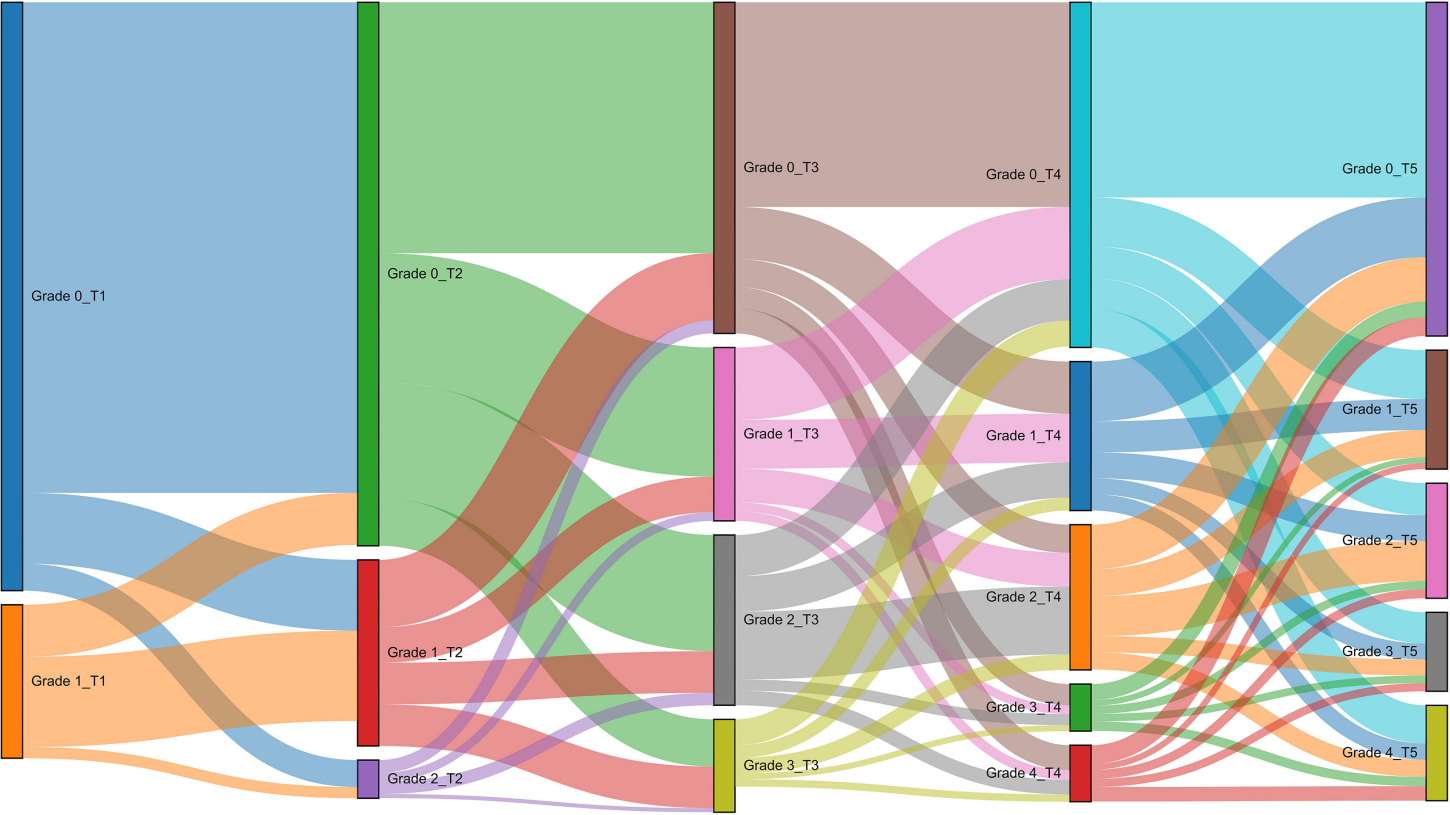
Analysis results of transfer between different severity grades in different periods. T1, T2, T3, T4, T5 represent stage 1, stage 2, stage 3, stage 4, and stage 5 respectively. Sankey diagram illustrating the flow of walnut trees between disease severity grades from T1 to T5. Node width is proportional to the proportion of trees in each grade; link width is proportional to the percentage transitioning between grades. T1–T5 = Stage 1 to Stage 5; Grade 0–4 severity classification as defined in Methods.

To investigate the progression of juglans leaf necrosis (JLN) across different severity levels and to characterize the transition from mild to severe infection, we constructed transition matrices between disease severity levels at different time points. These matrices were derived from the statistical analysis of 3.2 classification results in ArcGIS 10.8 and are used to quantify the dynamic changes in disease severity during the monitoring period. To illustrate the transitions between different severity classes visually, Sankey diagrams were employed. As shown in **Figure 9**, the diagrams clearly depict the flow of infected walnut trees between severity levels over time, providing intuitive insight into the development trajectory of JLN. This approach enables a better understanding of disease dynamics, highlighting the rates and directions of progression, persistence, or potential remission under field conditions.

From stage 1 to stage 2, 66.13% of the trees originally classified as severity grade 0 (healthy) remained stable, while 12.16% of those at grade 1 also maintained their status. Notably, 9.55% of trees transitioned from grade 0 to grade 1, and 3.64% advanced to grade 2. Additionally, 1.5% of trees at grade 1 progressed to grade 2. These findings indicate that the predominant trend during this stage was the early development of disease symptoms, primarily characterized by the progression from grade 0 to grade 1 and 2.

Between stage 2 and stage 3, the transition became more pronounced. Only 33.82% of grade 0 trees remained healthy, whereas the retention rates for grades 1 and 2 dropped significantly to 4.79% and 1.68%, respectively. Meanwhile, 17.41% of grade 0 trees progressed to grade 1, 15.67% to grade 2, and 6.37% to grade 3. Furthermore, 5.60% of grade 1 trees advanced to grades 2 and 3, while 0.54% of grade 2 trees transitioned to grade 3. This stage marked a rapid escalation in disease severity, with widespread movement toward higher severity levels.

From stage 3 to stage 4, the proportion of trees maintaining their original severity level continued to decline. Only 27.59% of grade 0 trees remained unchanged, followed by 6.58%, 9.15%, and 0.79% retention for grades 1, 2, and 3, respectively. Transitions from grade 0 to higher severity grades were observed at 7.02% (to grade 1), 3.79% (to grade 2), 2.88% (to grade 3), and 3.35% (to grade 4). Similarly, trees at grade 1 transitioned to grades 2 (4.55%), 3 (1.17%), and 4 (1.31%). Trees at grade 2 continued to progress, with 1.49% and 1.97% moving to grades 3 and 4, respectively, while 0.98% of grade 3 trees advanced to the most severe grade (grade 4). This stage reflected an acceleration in disease progression toward the advanced stages.

Between stage 4 and stage 5, disease advancement persisted, though at a slightly reduced rate. The stability rates for grades 0 through 4 were 26.31%, 6.57%, 9.15%, 0.79%, and 0.97%, respectively. Trees at grade 0 continued to show signs of infection, with 6.59%, 4.31%, 4.18%, and 5.13% transitioning to grades 1 through 4, respectively. Grade 1 trees progressed to grade 2 (3.47%), grade 3 (2.16%), and grade 4 (2.19%). Transitions from grade 2 to 3 (2.17%) and 4 (2.31%) were also observed, along with 1.25% of grade 3 trees reaching grade 4. These results indicate a steady but ongoing upward shift in disease severity, with increasing proportions of trees reaching moderate to severe grades in later stages.

## 4 Discussion

### 4.1 Vegetation indices and their role in disease detection

The use of vegetation indices based on hyperspectral data proved essential and effective in assessing the severity of JLN. In this study, greenness indices, leaf pigment indices, and canopy moisture or photosynthetic efficiency indices were calculated to capture different physiological aspects of plant health. Among them, greenness indices such as NDVI, EVI, and RENDVI effectively detected changes in overall canopy health. This is consistent with a study on chlorosis in the Shulayar Reserve Forest in Kerala, which showed that such indices are sensitive to vegetation vigor and chlorophyll content and can be used to monitor forest canopy health (Ahmad et al., 2020). Leaf pigment indices such as CRI1 and ARI1 can be used to identify early signs of stress related to chlorophyll degradation and carotenoid accumulation, which can indicate the presence of JLN. This aligns with findings that the chlorophyll-to-carotenoid ratio is a key indicator of physiological stress and that hyperspectral indices effectively track pigment changes during plant development (Song and Wang, 2022). Canopy moisture indices such as PRI and WBI were particularly valuable for assessing water stress commonly associated with JLN, providing additional insights into the underlying causes of the disease. Similar results were obtained in studies on Scots pine and sorghum, where PRI and red-edge-based indices reliably captured carotenoid activity and water stress dynamics (Miettinen et al., 2025; Song et al., 2023).

In addition, studies on strawberry and sweet corn also showed that chlorophyll fluorescence indices and red-edge-based vegetation indices (e.g., CIREDEDGE, REIP) are very effective in detecting the early stages of heat, water, and nitrogen stress, which strengthens the role of using hyperspectral indices to distinguish different types of physiological stress (Poobalasubramanian et al., 2022; Sellami et al., 2022). These findings also validate that combining multiple vegetation indices targeting chlorophyll, carotenoids, and water content can provide a comprehensive and early assessment of JLN. Future work could further refine this approach, as demonstrated in studies using deep learning and hyperspectral signatures to monitor tree and crop stress (Moley et al., 2024; Deng et al., 2023; Bai et al., 2024; Otone et al., 2024).

Our classification analysis showed that the combination of MRESRI, CRI1, and PRI achieved the highest performance across various metrics (classification accuracy, precision, recall, and Kappa coefficient). This highlights the synergistic value of integrating indices targeting different physiological traits. Studies on squash and tomato diseases using UAV hyperspectral imaging also supported the advantages of combined metrics for early disease stage classification, achieving more than 90% accuracy even under field conditions (Abdulridha et al., 2020a, Abdulridha et al., 2020b).

However, we found that misclassification occurred mainly between the “healthy” (0) and “mild” (1) categories, likely due to overlapping spectral signatures. This challenge was echoed in studies of wheat rust and rubber tree disease, where the early stages of the disease are the most difficult to distinguish, even with advanced models (Zeng et al., 2024; Abdulridha et al., 2023). We also found that the winds in the Hotan area are strong, and the leaves of walnut trees often have dust attached and accumulated, which can affect the accuracy of classification and also increases the difficulty of early disease monitoring. Despite these problems, the high accuracy in classifying “healthy” (0) and “severe” (3) categories, as well as strong overall performance, demonstrates that UAV hyperspectral imaging, especially when combined with field validation, remains a powerful and reliable method for disease monitoring in walnut orchards and other areas.

### 4.2 Disease progression and spatial–temporal dynamics

High-resolution UAV imagery and hyperspectral classification showed clear spatio-temporal progression of JLN. In the early stages (stages 1 and 2), visual symptoms of JLN were mild and difficult to detect with traditional remote sensing methods, including high-resolution RGB imagery. Slight textural changes in the canopy observed in RGB imagery indicate early stress, but symptoms were not obvious. This limitation is echoed by other studies that highlight the lack of spectral sensitivity of RGB imaging to detect physiological stress before it becomes apparent (Kouadio et al., 2023).

As the disease progresses to stage 3, visible symptoms such as browning and curling and scorching of canopy leaves became more evident. At this point, hyperspectral imaging became increasingly effective in capturing disease-related changes. A combination of vegetation indices (MRESRI, CRI1, and PRI) provided the best choice for classification, consistent with other studies in which hyperspectral imagery outperformed traditional methods in detecting disease progression. For example, hyperspectral sensors with neural networks achieved accurate classification of Huanglongbing in citrus by detecting early canopy reflectance anomalies (Deng et al., 2020). This further proves that hyperspectral remote sensing combined with physiological indicators can reliably detect and quantify the disease in early and intermediate stages, even before visible symptoms fully appear (Kuswidiyanto et al., 2022).

The study found that the spatial distribution of JLN in the study area also showed different patterns. The results showed that hotspots of JLN occurrence occurred more frequently near roads and low-lying areas. These areas may be affected by microclimatic conditions such as increased temperature, altered airflow, or poor drainage. Similar spatial patterns have been found in remote sensing studies of citrus and palm diseases, where the disease was found to be more severe near human infrastructure or waterlogged areas (Alsadik et al., 2024). This further validates the role of local environmental variables in driving disease onset and highlights the importance of drone data for spatially targeted monitoring.

In-depth analysis of the temporal dynamics of JLN (Section 3.4) showed that disease severity had a significant and rapid progression between early and late monitoring stages. Initially, more than 70% of trees were healthy or only slightly affected with indistinct symptoms (grades 0-1), but by stage 3, moderate symptoms (grade 2) became common and severe infections (grade 3) rose to nearly 9%, with affected walnut trees showing obvious symptoms and concentrated in patches. By stages 4 and 5, the proportion of trees showing severe to critical symptoms increased dramatically, indicating that JLN has the ability to intensify in a short period of time. Similar time series drone studies have revealed similar trends, with scholars using continuous hyperspectral monitoring and tracking of biotic stress in vineyards and wheat fields, finding that biotic stress in the study area also expanded rapidly in a short period of time (Nguyen et al., 2021; Zhang et al., 2025). Interestingly, during the development of JLN, new leaves will grow, and some tree crowns will show temporary recovery, which will mask the disease signal and reduce the detection accuracy. This phenomenon has also been observed in related studies when using chlorophyll fluorescence and solar-induced fluorescence index for stress monitoring. This physiological improvement may produce false negatives in automatic models (Chang et al., 2020). These findings emphasize the importance of continuous and high-frequency monitoring using drone hyperspectral, which can not only capture the trend of disease escalation but also potential mitigation or recovery trends.

### 4.3 Severity-level transitions and management implications

We used Sankey diagrams in Section 3.5 to illustrate the dynamic path of JLN from one severity level to another in the study area. During the early monitoring intervals (e.g., stage 1 to stage 2), most healthy trees (grade 0) remained in the same state, while only a small number of trees transitioned to grade 1 or 2. In contrast, in the mid-to-late intervals (stage 3 to stage 4 and stage 4 to stage 5), the severity accelerated significantly, with a considerable number of grade 1 and 2 trees progressing to grades 3-4. This pattern not only highlights the nonlinear progression of JLN but also indicates the importance of early management, where timely intervention management can prevent mild infection from evolving into large areas of burnt areas. Similar nonlinear disease progression patterns were observed in a drone-based maize study, where researchers used hyperspectral and wavelet features to capture disease transitions across time intervals. It was found that disease transitions at different levels in different periods were not linear, and the transitions were significantly accelerated in the later stages (Bai et al., 2024).

Notably, a small number of trees either remained stable in the mild category or transitioned downward from the moderately severe category to the mild category, a reversal that was often associated with new leaf growth. This reflects the heterogeneity of host responses, and some specific factors, such as genetic resilience or microclimate buffering, may attenuate or delay disease progression. This finding is consistent with the results of a time series study of *Fusarium solani* in cotton, which used deep learning and temporal modeling to explain changes in disease trajectories. The study found that some plants showed partial recovery or delayed symptom escalation during disease development (Abdalla et al., 2024).

For orchard managers, these findings imply that a one-size-fits-all approach to disease control may not be sufficient in orchard management and that more precise management, focused on “hotspots” or severely affected areas, is needed to produce cost-effective and timely results. This is consistent with recommendations from precision disease management studies that advocate the use of drones for spatial analysis and severity modeling, followed by site-specific precision interventions (Heidarian Dehkordi et al., 2020; Kouadio et al., 2023).

### 4.4 Limitations and challenges of UAV-based remote sensing

While drone-based hyperspectral imaging offers significant advantages for JLN monitoring, several limitations and challenges must be considered. A major limitation is the difficulty in accurately detecting early JLN due to subtle spectral differences between healthy and mildly infected trees. Even with optimized vegetation indices, distinguishing early symptoms from healthy canopies remains a challenge. This issue has also been noted in studies detecting early root rot effects on grapevines, where asymptomatic and diseased plants showed only slight spectral differences, and although machine learning approaches can improve classification accuracy, reflectance overlap between healthy states can lead to early misclassifications (Calamita et al., 2021).

Canopy complexity has also been found to complicate disease detection further. Heterogeneous canopies caused by mixed leaf ages, new sprouts, or overlapping branches can significantly distort spectral readings and increase classification errors. These challenges have been demonstrated in studies of oil palm diseases, where mixed classes within a single canopy often led to false positives, especially under shaded or partially obscured foliage (Anuar et al., 2021). Similarly, in apple orchards, studies have found that shadow pixels significantly reduce the accuracy of leaf area index (LAI) and chlorophyll content detection. Therefore, when using hyperspectral data for leaf area index (LAI) and chlorophyll content detection, shadow correction is required to reduce the error caused by it (Zhang et al., 2024).

Environmental conditions during drone flight (such as wind, light changes, and altitude changes) will also introduce noise into hyperspectral data sets, especially changes in light, which will affect the quality of hyperspectral data collection. Although the collected data requires further atmospheric correction and radiation calibration to reduce the errors caused by environmental conditions, the quality of the data may be limited by the constraints of on-site environmental conditions. This was also found in the citrus mold detection study, which showed that changing light conditions reduced the accuracy of the model during daytime flights, while the night vision-enhanced model provided more consistent results (Apacionado and Ahamed, 2023).

Although drones provide valuable insights, not all growers have access to this technology, especially those with limited financial resources or technical expertise. Similar concerns arise in a variety of crop systems, including viticulture and grassland management, where high-end sensors significantly outperform low-cost options but remain out of reach for most users (Hall and Lara, 2022). Therefore, reducing the cost and complexity of drone monitoring systems is critical for their wider adoption in precision agriculture.

## 5 Conclusion

In this study, we used UAV hyperspectral imaging and high-resolution visible light images combined with ground surveys to monitor juglans leaf necrosis (JLN) in walnut orchards in southern Xinjiang. We classified the different severities of JLN at different stages and analyzed its spatiotemporal distribution pattern characteristics to explore the occurrence and development of JLN. The main conclusions are as follows: The use of UAV high-resolution visible light images to study the occurrence and development of JLN provided limited by the limited bands and the complexity of the changes in JLN. Using hyperspectral images to calculate a variety of vegetation indices (especially MRESRI, CRI1 and PRI) with Random Forest achieved achieve fast and high-precision classification of JLN of different severity, which is an effective research method. Through the analysis of the development process of JLN, it was found that the process from mild to severe was rapid, highlighting the need for repeated, high-resolution monitoring. Spatial analysis further showed that juglans leaf necrosis (JLN) forms concentrated hotspots in low-lying areas, near roads, and areas with high tree density during its development, indicating that environmental factors affect disease distribution, and targeted management in these “hotspot” areas may help slow JLN progression. Our results highlight the practicality and scalability of drone-based remote sensing technology for large-scale orchard monitoring, providing orchard managers with timely insights to implement precise interventions. Future research should integrate other data sources to improve classification performance and develop predictive models to more proactively manage the development of JLN. By adopting and improving these remote sensing technologies, growers can reduce yield losses, improve resource allocation, carry out ecological protection, and ultimately promote the sustainable development of Xinjiang’s walnut industry.

## Data Availability

The original contributions presented in the study are included in the article/supplementary material. Further inquiries can be directed to the corresponding author.
